# Catheter-associated Urinary Tract Infections—Online Questionnaire: Status Quo in Central European Urological Management of Catheter-associated Urinary Tract Infection

**DOI:** 10.1016/j.euros.2024.08.018

**Published:** 2024-09-16

**Authors:** Emilio Arbelaez, Iris Zünti, Sarah Tschudin-Sutter, Andreas Zeller, Florian S. Halbeisen, Hans-Helge Seifert, Kathrin Bausch

**Affiliations:** a Department of Urology, University Hospital Basel, Basel, Switzerland; b University of Basel, Basel, Switzerland; c Division of Infectious Diseases and Hospital Epidemiology, University Hospital Basel, Basel, Switzerland; d University Centre for Primary Health Care, University of Basel, Basel, Switzerland; e Surgical Outcome Research Center, Department of Clinical Research, University Hospital Basel and University of Basel, Basel, Switzerland

**Keywords:** Antimicrobial resistance, Antimicrobial stewardship, Catheter, European, Guidelines, Urinary tract infection

## Abstract

**Background and objective:**

Catheter-associated urinary tract infections (CAUTIs) represent a significant burden in health care and its management is challenging. This study aims to assess and compare central European CAUTIs regarding diagnostics, treatment, and prophylaxis.

**Methods:**

An anonymized online questionnaire was distributed among urologists in Austria, France, Germany, and Switzerland between January and October 2023, consisting of demographic questions on catheter management and diagnostics, treatment, and prophylaxis of CAUTIs. An analysis was performed per country. Comparisons were done with the Fisher’s exact test (statistical significance with *p <* 0.05).

**Key findings and limitations:**

Out of 423 participating urologists, most regularly performed catheter changes and managed catheter-related issues, except for French urologists. Swiss urologists tended to change the catheter after a longer interval. In France, a higher estimated number of CAUTIs were observed. Diagnostic symptoms and measures varied significantly between countries. French urologists prescribed more antimicrobials per patient and administered longer treatment regimens. The choice of antimicrobial agents differed notably for nonfebrile and febrile CAUTIs, with cotrimoxazole/nitrofurantoin being common for nonfebrile cases and cephalosporin/amoxicillin for febrile ones. Follow-up protocols were similar among urologists, while prophylactic measures showed variations.

**Conclusions and clinical implications:**

CAUTI management varied notably across countries in terms of diagnostics, treatment, and prophylaxis. Discrepancies in antimicrobial therapy could be influenced by local resistance rates; yet, nonrecommended drugs and prolonged regimens, as compared with guideline recommendations, were common. This trend, along with inappropriate diagnostics and prophylaxis, may increase antimicrobial resistance and CAUTI morbidity. This study emphasizes the necessity for diagnostic and antimicrobial stewardship interventions, and proper training in CAUTI management.

**Patient summary:**

In this questionnaire-based study examining the clinical practices for managing urinary tract infections in patients with bladder catheters (CAUTIs), significant disparities were observed among European urologists regarding diagnosis, treatment, and prophylaxis. These findings underscore the critical need for clear guidelines and comprehensive training in CAUTI management.

## Take Home Message

Cather-associated urinary tract infection management varies among European countries, with nonrecommended antimicrobials and prolonged treatments being used commonly. This may worsen antimicrobial resistance and morbidity. Urgent actions, including diagnostic and antimicrobial stewardship, guideline adherence, and enhanced training in the management of catheter-associated urinary tract infections, are needed.

## 1 Introduction

Catheter-associated urinary tract infections (CAUTIs) represent a significant burden in health care, being one of the most prevalent nosocomial infections at 32.2–40% [1]. Approximately one-fourth of all hospitalized patients [2] and around 5% of patients in nursing homes [3] require a urinary catheter. Around 70–80% of urinary tract infections (UTIs) are associated with catheter use [4]. The implications of CAUTIs are far reaching, often resulting in extended hospital stays and elevated morbidity and mortality [5], [6].

In response to this health care challenge, considerable efforts have been directed toward minimizing the incidence of CAUTIs. The primary strategy has been to reduce the duration of catheterization, which is the main risk factor for CAUTIs [7], [8], [9]. However, this approach is not universally applicable due to a significant subset of patients necessitating long-term catheterization. This population, often characterized by additional risk factors for UTIs such as advanced age, immobilization, diabetes, or immunosuppression, presents a unique challenge in CAUTI management [10], [11].

However, management of CAUTIs presents a myriad of challenges, spanning from symptoms and diagnosis to treatment and prophylaxis [12], [13], [14], [15]. Characteristic symptoms of UTIs, such as urinary frequency and dysuria, are usually absent, leading to a reliance on atypical and heterogeneous symptoms such as catheter obstruction [16], abdominal pain, fever, and general health deterioration [17]. Diagnostic efforts are further complicated by biofilm formation [18] and bacterial colonization or asymptomatic bacteriuria (ABU) [19]. Causative bacteria are frequently multiresistant [20]. Current guidelines recommend treating CAUTIs as complicated UTIs, necessitating extended antibiotic treatment, while CAUTI-specific prophylactic measures remain largely unexplored [13].

To establish a foundation for future antimicrobial stewardship programs and recommendations, our study aimed to investigate the diagnosis, treatment, and prophylactic management of CAUTIs among urologists in central Europe, thereby assessing the current clinical routine.

## 2 Materials and methods

### 2.1 Study design and setting

We conducted an international, web-based, anonymous survey among urologists in Austria, France, Germany, and Switzerland. From January 2023 onward, the REDCap-based survey was distributed through the networks of the Austrian (Österreichische Gesellschaft für Urologie), French (Association Française d'Urologie), German (Deutsche Gesellschaft für Urologie and Berufsverband der deutschen Urologie), and Swiss (Schweizerische Gesellschaft für Urologie) associations of urology by e-mail. The contacted physicians were asked to additionally distribute the questionnaires to urologists in their institutions. A reminder of participation was sent 2 wk later.

The survey included six demographic questions, three questions on catheter management in general, and 13 questions on CAUTIs. The majority of questions had given answers to choose from. In some, only individual answers were possible and in others multiple answers (see the Supplementary material). The detailed questionnaire was provided in German, French, and Italian. The English version can be found in the Supplementary material. After collection of baseline characteristics, respondents were asked whether they were involved in catheter management. If this question was answered with “no,” the subsequent questions were not asked. Participants could only proceed to the next question if the previous question was answered. Questionnaires that were not completed fully were also included in the analysis.

Participation in the survey was voluntary, and no identifiers and written informed consent were collected from respondents to ensure anonymity. Ethics committee approval was not required for this study, as the survey was directed to urologists, and no patient data were gathered.

### 2.2 Statistical analysis

We conducted comprehensive descriptive statistical analyses to summarize the characteristics of the collected data. For categorical variables, we provided the frequency and corresponding proportions. For continuous variables, we selected appropriate descriptive statistics based on the nature of data distribution. Owing to the non-normal nature of the data, we used median and range to report.

To examine differences in the study population characteristics between countries, we employed the Kruskal-Wallis rank-sum test for continuous variables. For categorical variables, Fisher’s exact test was used to determine the presence of any significant differences in the proportions across different countries. Further, we performed Fisher’s exact test separately for each question in the survey, which allowed us to identify country-specific variations in responses.

We considered a *p* value of <0.05 to be statistically significant. All statistical analyses were performed using the R statistical software (version 4.2.2; The R Foundation for Statistical Computing, Vienna, Austria).

## 3 Results

In total, 423 questionnaires were completed, out of which 84.4% were completed fully. Fifteen participants were not involved in catheter management and were excluded from the analysis. Seventy-eight Austrian, 126 French, 134 German, and 85 Swiss urologists were included in the study analysis. The median age was 53 yr (range, 27–80). Urologists’ basic characteristics differed significantly and are displayed in Table 1.

**Table 1:** Characteristic table

Characteristic	Austria (*N* = 78)		France (*N* = 8126)		Germany (*N* = 134)		Switzerland (*N* = 85)		*p* value
	*N*	%	*N*	%	*N*	%	*N*	%	
Age, median (range)	45.5	27–77	51	31–75	55	27–80	48	28–70	<0.001
Years since working in medical profession, median (range)	20	1–50	24	5–333	27	1–50	20	2–45	0.003
Sex									<0.001
Female	29	38.16	13	10.74	25	19.23	22	26.83	
Male	47	61.84	108	89.26	105	80.77	60	73.17	
Medical facility where you are working									<0.001
Urological practice	27	35.53	54	44.63	111	85.38	37	45.12	
District hospital	23	30.26	38	31.4	2	1.54	9	10.98	
Cantonal hospital	12	15.79	8	6.61	6	4.62	21	25.61	
University hospital	12	15.79	20	16.53	9	6.92	8	9.76	
Rehabilitation hospital	2	2.63					4	4.88	
Others			1	0.83	2	1.54	3	3.66	
Do you look after patients who are permanently supplied with a urinary catheter?									<0.001
No					1	0.77			
Rarely			13	10.74			1	1.22	
Yes	2	2.63	9	7.44	2	1.54			
Yes and I change transurethral	4	5.26	12	9.92					
Yes and I change suprapubic	1	1.32	2	1.65	1	0.77			
Yes and I change both	69	90.79	85	70.25	126	96.92	81	98.78	
At what interval do you usually perform catheter changes in asymptomatic patients?									<0.001
<2 wk					1	0.78	1	1.22	
2–4 wk	7	9.21	8	6.61	14	10.85	2	2.44	
1–2 mo	65	85.53	84	69.42	108	83.72	41	50	
2–3 mo	3	3.95	22	18.18	6	4.65	38	46.34	
>3 mo	1	1.32	2	1.65					
Only if needed			5	4.13					
During the past 12 mo, how many patients with transurethral and/or suprapubic catheters did you see on average per week for catheter-related concerns?									<0.001
<1	6	7.89	54	44.63	5	3.88	10	12.2	
1–5	32	42.11	52	42.98	32	24.81	34	41.46	
5–10	24	31.58	10	8.26	43	33.33	28	34.15	
11–25	9	11.84	4	3.31	33	25.58	9	10.98	
26–50	5	6.58	1	0.83	13	10.08			
>50					3	2.33	1	1.22	
If you estimate, how often do you diagnose a UTI in a catheterized patient per year?									<0.001
Fewer than once	6	8.11	9	7.69	14	11.11	14	18.18	
Once per year	17	22.97	12	10.26	26	20.63	22	28.57	
2–3 times per year	21	28.38	24	20.51	44	34.92	19	24.68	
4–5 times per year	7	9.46	18	15.38	14	11.11	2	2.6	
>5 times per year	23	31.08	54	46.15	28	22.22	20	25.97	
Do you feel competent in managing catheters and recurrent urinary tract infections in catheterized patients?									<0.001
Rather no			2	2.13	2	1.65			
Rather yes	15	21.74	59	62.77	27	22.31	24	32.88	
Yes	54	78.26	33	35.11	92	76.03	49	67.12	

UTI = urinary tract infection.

Fully answered questionnaires: 84.4%.

Intervals between regular catheter changes differed significantly between countries (Table 1). French urologists treated patients with transurethral and/or suprapubic catheters less frequently than the other urologists. Urologists in Austria and France diagnosed UTIs per catheterized patient per year more often than those in the other countries (Table 1).

French questionees felt less competent in managing CAUTIs than urologists in the other countries (*p* < 0.001; Table 1).

**Fig. 1:**
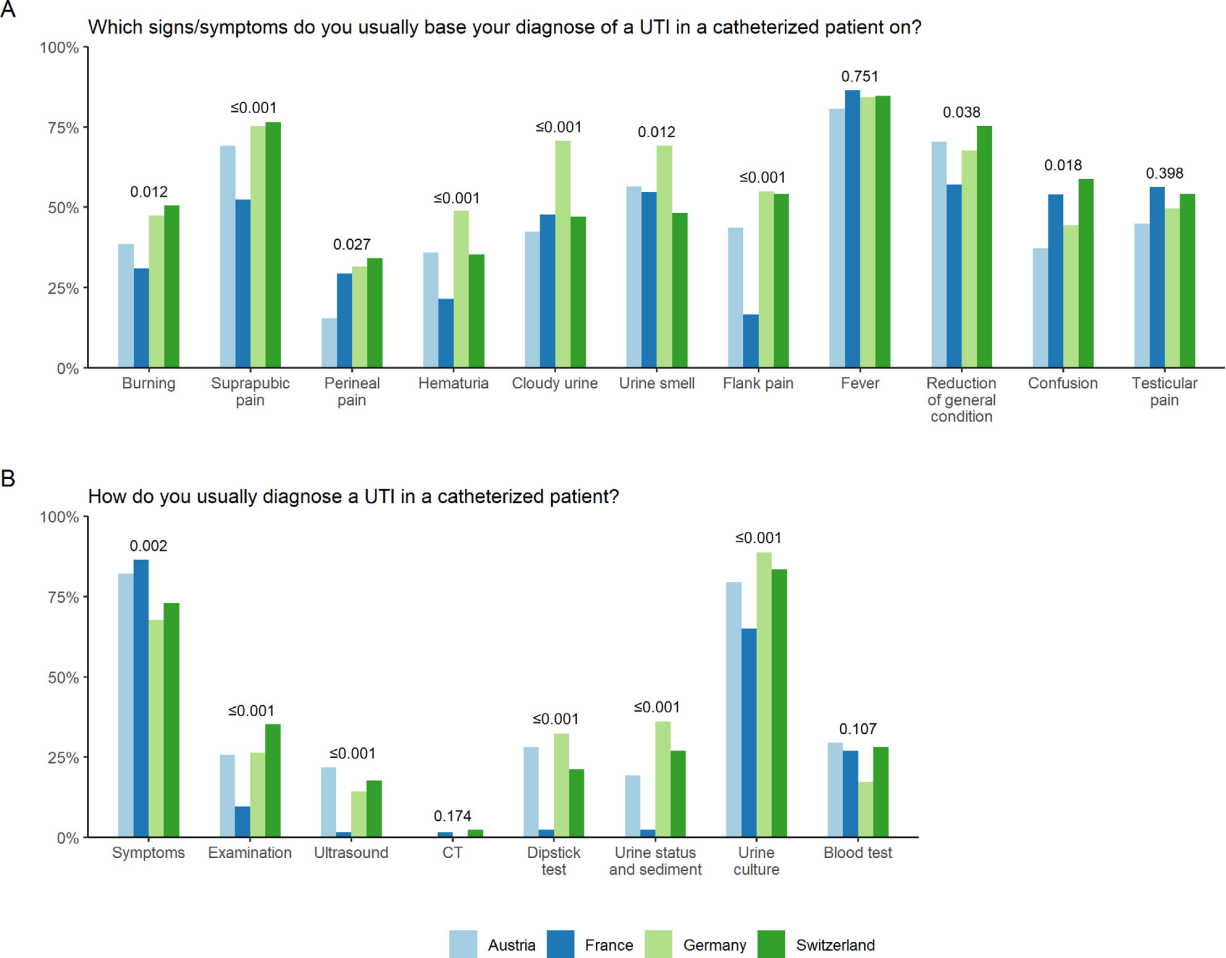
Diagnosis of CAUTI. Answers are depicted per country (light blue, Austria; dark blue, France; light green Germany; dark green Switzerland). CAUTI = catheter-associated urinary tract infection; CT = computed tomography; UTI = urinary tract infection.

Figure 1 shows responses regarding CAUTI diagnostics and compares the respective countries. In total, the symptoms that urologists based their diagnosis on varied between countries (Fig. 1A). For fever and testicular pain, no statistically significant difference could be shown (*p* = 0.751 and *p* = 0.398, respectively; Fig. 1A). Cloudy urine and smell were frequently taken as diagnostic symptoms (eg, up to 75% in Germany; Fig. 1A). Most frequently chosen measures for CAUTI diagnosis were symptoms and urine culture. Between countries, measures chosen varied statistically significantly except for computed tomography (*p* = 0.174), which was barely performed on a routine basis, and blood test (*p* = 0.107; Fig. 1A).

**Fig. 2:**
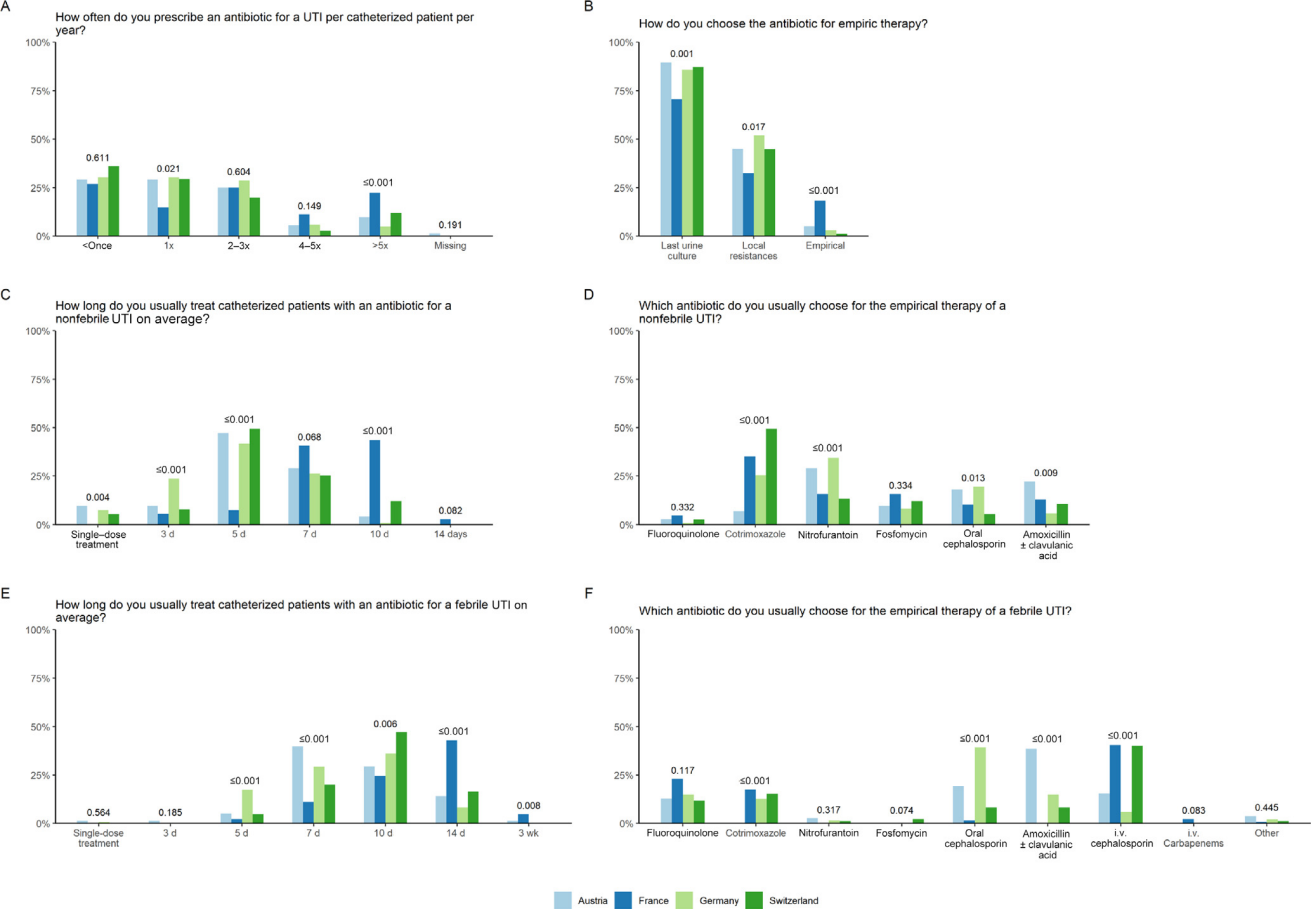
Treatment of CAUTI. Answers are depicted per country (light blue, Austria; dark blue, France; light green Germany; dark green Switzerland). CAUTI = catheter-associated urinary tract infection; i.v. = intravenous; UTI = urinary tract infection.

Regarding CAUTI treatment, the majority of urologists stated to treat a catheterized patient fewer than once per year. French urologists stated to treat more patients more often (ie, more than five times) with antibiotics than the urologists of the other countries (*p* < 0.001; Fig. 2A).

Antibiotic therapy was chosen mainly by the last urine culture (Fig. 2B). French urologists chose empirical treatment more often than the other urologists (*p* < 0.001; Fig. 2B). Antibiotic therapy was applied mainly for 5–7 d in nonfebrile patients and for 7–10 d in febrile CAUTI patients (Fig. 2C and 2E). In both, longer treatment was chosen in France (Fig. 2C and 2E). Figures 2D and 2F show the choice of antibiotic treatment in nonfebrile and febrile CAUTI patients.

**Fig. 3:**
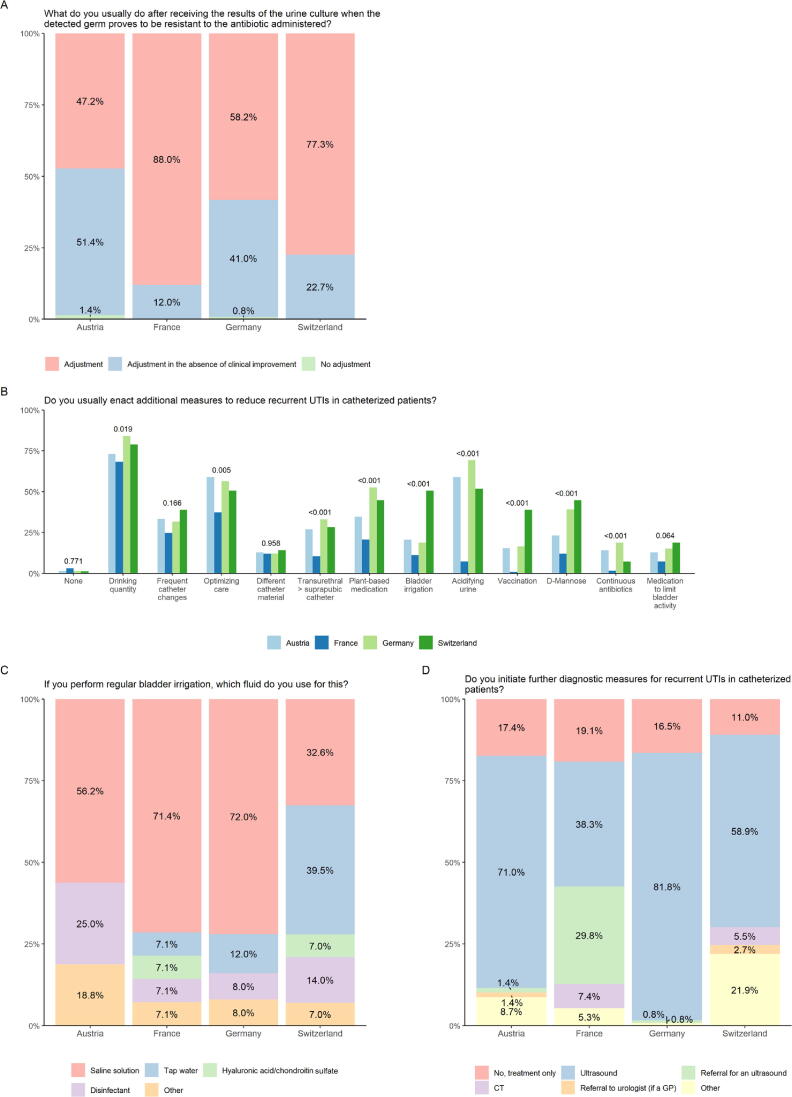
Prophylaxis of CAUTI. Answers are depicted per country (light blue, Austria; dark blue, France; light green Germany; dark green Switzerland). CAUTI = catheter-associated urinary tract infection; CT = computed tomography; GP = general practitioner; UTI = urinary tract infection.

After empiric treatment, most questionees would adjust the administered antibiotic according to urine culture. Except in France, many urologists change the antibiotic only if there is no clinical improvement (Fig. 3A and Supplementary Table 1). Figure 3B demonstrates various prophylactic measures. The most frequently chosen measure was an increase of drinking quantity. The use of bladder irrigation is executed mainly with saline solution except in Switzerland, where it is common to use tap water (Fig. 3C and Supplementary Table 2). Regarding further diagnostics in recurring CAUTIs, ultrasound is used mainly as an adjunct, as shown in Figure 3D (Supplementary Table 3).

## 4 Discussion

This study is the first status quo assessment of urological CAUTI management in central European countries. It underlines vast inhomogeneity in diagnosis, treatment, and prophylaxis of CAUTIs.

Potentially, this inhomogeneity results from different training and decision-making of urologists in the countries considered. One important basis for decision-making is guideline recommendations.

Urologists in Austria, Germany, and Switzerland make their decisions primarily based on the guidelines of the European Association of Urology (EAU), which include a summary of literature on CAUTI management [13]. Even though there are UTI guidelines in Germany [21], these are not CAUTI specific. Only the French Urological Association (AFU) provides guidelines on CAUTIs [14]. The European and French recommendations are similar but differ in certain details: the EAU suggests changing the catheter before obtaining a urine culture, while the AFU advises getting the culture from the indwelling catheter and changing it 24 h after starting antimicrobial treatment. AFU guidelines are strict on antimicrobial selection being based on the infectious focus (eg, cystitis and prostatitis), while the EAU suggests considering local resistances and categorizes CAUTIs as complicated UTIs, recommending corresponding treatment [13], [14]. Comparison of AFU and EAU guidelines showed longer treatment durations in the former, but further CAUTI management was not analyzed in this study [22]. This underlines our findings, observing a longer treatment duration among French urologists than in those of the other countries. Several studies have demonstrated the importance of guideline adherence, leading to a more judicious use of antimicrobials and decreased resistance rates [23], [24].

However, good clinical practice is based not only on guideline adherence, but also on clinical routine. Our findings indicate that French urologists are less involved in catheter management and express more insecurity in managing CAUTIs. This may be attributed to the preference for self-catheterization over indwelling catheterization in France [25]. Other reasons might include the fact that, at least in inpatient settings in France, antibiotic therapies are often determined by infectious disease specialists. Whether this is a reason for potential differences would need to be investigated separately.

Despite general differences, we find certain similarities in diagnostics such as relying diagnosis on symptoms and culture, which is in line with the EAU and Center for Disease Control and Prevention guidelines [13], [15].

Surprisingly, >25% of urologists in Austria, Germany, and Switzerland base diagnosis on dipstick and urine sediment even though these measures are highly unspecific in the context of CAUTIs due to the high incidence of ABU, pyuria, or microhematuria in catheterized patients [13], [24], [25].

A positive urine culture alone does not imply a CAUTI. In the absence of symptoms, it should always be considered an ABU, which is common in catheterized patients due to catheter colonization. Screening and treating ABU in these patients does not provide any benefit [24], [26]. This is generally a weak point in CAUTI literature and explains possible uncertainties in CAUTI recommendations and management: many studies often describe catheter-associated bacteriuria only and do not differentiate between catheter-associated ABU and CAUTI. Some studies use the term CAUTI when describing catheter-associated ABU [24].

This underlines the importance of symptom assessment for diagnosis, although in practice it is challenging in CAUTI patients due to their presentation with rather atypical symptoms (eg, rigors, altered mental status, and malaise/lethargy) [13]. This may explain why our study observed many differences in terms of symptoms between the countries. Surprisingly, among all urologists, a smelly and cloudy urine was frequently chosen to be a diagnostic symptom, even though it is mentioned explicitly in both EAU and AFU guidelines that an odorous or cloudy urine is neither a sign for a CAUTI nor a sign for a UTI [13], [14], [24].

French urologists prescribed antibiotics significantly more often. Furthermore, the antimicrobials chosen were less frequently based on the previous culture but more often empirically. This is potentially based on the more concrete recommendations for empiric treatment in France [14]. The duration of antimicrobial treatment was also significantly longer for nonfebrile and febrile CAUTIs among French urologists. German urologists, however, treated nonfebrile CAUTIs in a significantly shorter time, even though 7 d are recommended by the EAU [13]. We hypothesize that German urologists may view a CAUTI as an uncomplicated UTI, leading to non–guideline-conforming treatment. This might also explain the frequent use of nitrofurantoin in Germany, despite it not being recommended for complicated UTIs such as CAUTIs [21]. On the contrary, AFU guidelines differentiate CAUTIs based on the focus and severity of primary infection, impacting treatment duration and choice of antimicrobial. This suggests that the appropriate therapy regimen and duration may vary depending on these factors. Even if this can be supported by studies on treatment duration in different UTI manifestations (eg, cystitis and prostatitis), there is no CAUTI-specific literature on this [24], [27], [28].

Observed differences in antibiotic substances could furthermore be explained by different local antibiotic resistances. Resistance data are often not published consistently or accessible easily for prescribing practitioners [29], [30], [31], [32], [33], [34]. The resistance rate for amoxicillin among *Escherichia coli* in Europe is significantly high, warranting reconsideration of its use as empirical therapy for UTIs, as we observed in Austria [13], [29], [31], [32], [33], [34]. Taking into consideration that CAUTIs are usually caused by more complex bacteria (eg, different germs and multiple resistances) and affect a more frail patient population, empiric treatment needs to be highly efficient [10], [11].

One of the principles of antibiotic stewardship is to respect guidelines; however, one could criticize about EAU guidelines that these are not concrete on treatment duration and substance, but refer to complicated UTI guidelines only [13]. The differences between countries regarding treatment duration and substances can be well explained by this, and furthermore, it complicates the implementation of antimicrobial stewardship programs.

Following treatment with a resistant substance, Austrian, German, and Swiss urologists frequently adjusted the therapy only in the absence of clinical improvement. This is against the principles of antimicrobial stewardship as an antibiotic without any proven effect is administered longer than needed, which might result in early reinfection and selection of antimicrobial resistance [35], [36], [37].

To this date, there are no recommended prophylactic measures specific to CAUTIs, which is reflected in the inhomogeneity of our results [13], [14], [38]. Only increasing drinking quantity, selection of the catheter material, as well as frequent catheter changes were responded homogenously. Bladder irrigation is commonly performed as the standard management of long-term urinary catheters, but it remains a controversial method. A Cochrane review based on studies with poor methodological quality found inconclusive evidence for the role of bladder irrigation in preventing CAUTIs [39]. Even though not recommended by guidelines, bladder irrigation (with tap water) is used frequently in Switzerland. A recent study showed that bladder irrigation with tap water reduced CAUTI occurrence and antibiotic use [40]. Studies and guideline recommendations on CAUTI-specific prophylactic measures are highly needed.

One limitation of our study was that it was not based on direct observations, which did not allow accounting for recall and reporting biases. Further, our survey results may not adequately represent CAUTI practices among Austrian, French, German, and Swiss urologists, as the overall response rate cannot be reproduced and was potentially low. However, it is conceivable that compliance with CAUTI guidelines in nonrespondents is not considerably higher than in those respondents who are less interested in this topic.

Lastly, CAUTI literature is highly heterogeneous, and even if the recommendations are classified as strong, many sensible clinical questions are not addressed. Nevertheless, the attempt to find possible reasons for the described differences is often purely speculative and highlights the urgent need for clear guideline recommendations and training in the management of CAUTIs.

## 5 Conclusions

There are significant variations in the management of CAUTIs across central European countries, encompassing diagnostics, treatment, and prophylaxis. Discrepancies in antimicrobial treatment can be influenced by local antimicrobial resistance rates. However, the frequent use of nonrecommended antimicrobials or prolonged treatment regimens compared with current guidelines could escalate the rates of antimicrobial resistance and CAUTI-related morbidity. This study underscores the necessity for diagnostic and antimicrobial stewardship interventions, additional clinical trials to refine guideline recommendations, and proper training in CAUTI management.

  ***Author contributions*:** Kathrin Bausch had full access to all the data in the study and takes responsibility for the integrity of the data and the accuracy of the data analysis.

  *Study concept and design*: Arbelaez, Bausch, Zeller, Tschudin-Sutter.

*Acquisition of data*: Arbelaez, Bausch.

*Analysis and interpretation of data*: Halbeisen, Arbelaez, Bausch.

*Drafting of the manuscript*: Arbelaez, Bausch, Zünti.

*Critical revision of the manuscript for important intellectual content*: Seifert, Zeller, Tschudin-Sutter.

*Statistical analysis*: Halbeisen.

*Obtaining funding*: None.

*Administrative, technical, or material support*: Arbelaez, Halbeisen.

*Supervision*: Bausch, Seifert.

*Other*: None.

  ***Financial disclosures:*** Kathrin Bausch certifies that all conflicts of interest, including specific financial interests and relationships and affiliations relevant to the subject matter or materials discussed in the manuscript (eg, employment/affiliation, grants or funding, consultancies, honoraria, stock ownership or options, expert testimony, royalties, or patents filed, received, or pending), are the following: None.

  ***Funding/Support and role of the sponsor*:** None.
