# Supplementary material for: Catheter-associated Urinary Tract Infections—Online Questionnaire: Status Quo in Central European Urological Management of Catheter-associated Urinary Tract Infection
Source: Eur Urol Open Sci. 2024 Sep 16;69:63–70. doi: 10.1016/j.euros.2024.08.018 (PMC11421338; doi:10.1016/j.euros.2024.08.018)
Supplement: Supplementary Data 1 [file mmc1.docx]

**Supplementary material**

**Questionnaire**

Questions and answer options in brackets

^a^ single choice

^b^ multiple choice

In which country are you working? (Austria, France, Germany, Switzerland)^a^

Age (free text)

Years since working in medical profession (free text)

Sex (female, male)^a^

Years since working in medical profession (free text)

Medical facility where you are working (urological practice, district hospital, cantonal hospital, university hospital, rehabilitation hospital, others)^a^

Do you look after patients who are permanently supplied with a urinary catheter? (no, rarely, yes, yes and I change transurethral, yes and I change suprapubic, yes and I change both)^a^

At what interval do you usually perform catheter changes in asymptomatic patients? (< 2 weeks, 2-4 weeks, 1-2 month, 2-3 month, > 3 month, only if needed)^a^

During the past 12 months, how many patients with transurethral and/or suprapubic catheters did you see on average per week for catheter-related concerns? (< 1, 1-5, 5-10, 11-25, 26-50, >50)^a^

If you estimate, how often do you diagnose a UTI in a catheterized patient per year? (<once/year, once/year, 2-3/year, 4-5/year, >5/year)^a^

Do you feel competent in managing catheters and recurrent urinary tract infections in catheterized patients? (rather no, rather yes, yes)^a^

On which signs/symptoms do you usually base your diagnosis of a UTI in a catheterized patient? (burning, suprapubic pain, perineal pain, hematuria, cloudy urine, urine smell, flank pain, fever, reduction of general condition, confusion, testicular pain) ^b^

How do you usually diagnose a UTI in a catheterized patient? (symptoms, examination, ultrasound, CT, dipstick test, urine status and sediment, urine culture, blood test)^b^

How often do you prescribe an antibiotic for a UTI per catheterized patient per year? (< once, 1x, 2-3x, 4-5x, >5x)^a^

How do you choose the antibiotic for empiric therapy? (last urine culture, local resistances, empirical)^b^

How long do you usually treat catheterized patients with an antibiotic for a non-febrile UTI in average? (single-dose treatment, 3 days, 5 days, 7 days, 10 days, 14 days)^a^

Which antibiotic do you usually choose for the empirical therapy of a non-febrile UTI? (Fluorchinolone, Cotrimoxazole, Nitrofunantoin, Fosfomycine, oral Cephalosporine, Amoxicilline +/- clavulanic acid)^a^

How long do you usually treat catheterized patients with an antibiotic for a febrile UTI in average? (single-dose treatment, 3 days, 5 days, 7 days, 10 days, 14 days, 3 weeks)^a^

Which antibiotic do you usually choose for the empirical therapy of a febrile UTI? (Fluorchinolone, Cotrimoxazole, Nitrofunantoin, Fosfomycine, oral Cephalosporine, Amoxicilline +/- clavulanic acid, i.v. Cephalosporine, i.v. Carbapenems, other)^a^

What do you usually do after receiving the results of the urine culture when the detected germ proves to be resistant to the antibiotic administered? (adjustment, adjustment in the absence of clinical improvement, no adjustment)^a^

Do you usually enact additional measures to reduce recurrent UTIs in catheterized patients? (none, drinking quantity, frequent catheter changes, optimizing care, different catheter material, transurethral -> suprapubic catheter, plant-based medication, bladder irrigation, acidifying urine, vaccination, D-Mannose, continuous antibiotics, medication to limit bladder activity)^b^

If you perform regular bladder irrigation, which fluid do you use for this? (saline solution, tap water, hyaluronic acid/chondroitin sulphate, disinfectant, others)^a^

Do you initiate further diagnostic measures for recurrent UTIs in catheterized patients? (no, treatment only, ultrasound, referral for an ultrasound, CT, referral to urologist (if a GP), other) ^a^

Supplementary Table 1

What do you usually do after receiving the results of the urine culture when the detected germ proves to be resistant to the antibiotic administered?^a^

|  |  | **N Total** | **Austria** | **%** | **France** | **%** | **Germany** | **%** | **Switzerland** | **%** | **P-value** |
| --- | --- | --- | --- | --- | --- | --- | --- | --- | --- | --- | --- |
|  | Adjustment | 258 | 34 | 47.2 | 95 | 88 | 71 | 58.2 | 58 | 77.3 | <0.001 |
|  | Adjustment in the absence of clinical improvement | 117 | 37 | 51.4 | 13 | 12 | 50 | 41 | 17 | 22.7 |  |
|  | No adjustment | 2 | 1 | 1.4 | 0 | 0 | 1 | 0.8 | 0 | 0 |  |

Supplementary Table 2

If you perform regular bladder irrigation, which fluid do you use for this? ^a^

|  |  | **N Total** | **Austria** | **%** | **France** | **%** | **Germany** | **%** | **Switzerland** | **%** | **P-value** |
| --- | --- | --- | --- | --- | --- | --- | --- | --- | --- | --- | --- |
|  | Saline solution | 51 | 9 | 56.2 | 10 | 71.4 | 18 | 72 | 14 | 32.6 | 0.011 |
|  | Tap water | 21 | 0 | 0 | 1 | 7.1 | 3 | 12 | 17 | 39.5 |  |
|  | Hyaluronic acid/chondroitin sulphate | 4 | 0 | 0 | 1 | 7.1 | 0 | 0 | 3 | 7 |  |
|  | Disinfectant | 13 | 4 | 25 | 1 | 7.1 | 2 | 8 | 6 | 14 |  |
|  | Other | 9 | 3 | 18.8 | 1 | 7.1 | 2 | 8 | 3 | 7 |  |

Supplementary Table 3

Do you initiate further diagnostic measures for recurrent UTIs in catheterized patients? ^a^

|  |  | **N Total** | **Austria** | **%** | **France** | **%** | **Germany** | **%** | **Switzerland** | **%** | **P-value** |
| --- | --- | --- | --- | --- | --- | --- | --- | --- | --- | --- | --- |
|  | No, treatment only | 58 | 12 | 17.4 | 18 | 19.1 | 20 | 16.5 | 8 | 11 | <0.001 |
|  | Ultrasound | 227 | 49 | 71 | 36 | 38.3 | 99 | 81.8 | 43 | 58.9 |  |
|  | Referral for an ultrasound | 30 | 1 | 1.4 | 28 | 29.8 | 1 | 0.8 | 0 | 0 |  |
|  | Computed tomography | 11 | 0 | 0 | 7 | 7.4 | 0 | 0 | 4 | 5.5 |  |
|  | Referral to urologist (if a GP) | 3 | 1 | 1.4 | 0 | 0 | 0 | 0 | 2 | 2.7 |  |
|  | Other | 28 | 6 | 8.7 | 5 | 5.3 | 1 | 0.8 | 16 | 21.9 |  |
